# Continuous ventilation versus 30:2 strategy in mechanical cardiopulmonary resuscitation – a manikin-based simulation study

**DOI:** 10.1186/s12873-026-01493-z

**Published:** 2026-02-11

**Authors:** Bernhard Benda, Oliver Fuchs, Magdalena Benda, Fabian Perschinka, Nicolas Prokes, Thomas Ploner, Andrea Köhler, Michael Joannidis, Frank Hartig

**Affiliations:** 1 Core Facility Internal Emergency and Intensive Care Medicine, University Clinic Innsbruck, Innsbruck, Austria; 2 Department of Internal Medicine, Kufstein Hospital, Kufstein, Austria; 3 Department of Internal Medicine II, Academic Teaching Hospital, Feldkirch, Austria

**Keywords:** Mechanical chest compression device (MCD), Cardiopulmonary resuscitation (CPR), 30:2, Continuous ventilation (CV), Resuscitation, Mechanical CPR (mCPR)

## Abstract

**Background:**

There is conflicting data about which ventilation strategy is better when performing external chest compressions with mechanical devices. During manual cardiopulmonary resuscitation, continuous ventilation is recommended when the airway is secured and a ventilation rate of 10/min is maintained. However, during mechanical cardiopulmonary resuscitation, some expert groups and various data have raised concerns about ineffective ventilation with continuous ventilation due to the high intrathoracic pressure generated by the devices without pauses for effective ventilation. This study compares the two possible ventilation strategies - continuous ventilation and 30:2 (30 compressions followed by 2 ventilations) - during mechanical cardiopulmonary resuscitation. We analysed the effects of the two resuscitation strategies on ventilatory parameters measured during cardiopulmonary resuscitation and compared mechanical with manual cardiopulmonary resuscitation with both ventilation strategies.

**Methods:**

30 students of human medicine performed cardiopulmonary resuscitation on endotracheally intubated resuscitation training manikins. The chest compressions were performed manually or by using mechanical chest compression devices (LUCAS^®^, EASY PULSE^®^). Tidal volume, respiratory minute ventilation volume, ventilation frequency, compression frequency, and chest compression depth were recorded by sensors built into the manikins. Cardiopulmonary resuscitation was performed with either continuous ventilation (ventilations every 6 s; 10/min) or 30:2 in two resuscitation runs of 3 min each.

**Results:**

Respiratory minute ventilation volume was significantly higher with continuous ventilation than with 30:2 cardiopulmonary resuscitation (4.8 l/min vs. 3.1 l/min, *p*<.001). Tidal volume was lower with continuous ventilation than with a 30:2 cardiopulmonary resuscitation (521 ml vs. 565 ml; *p* = .013). There were no significant differences between manual and mechanical cardiopulmonary resuscitation with both ventilation strategies.

**Conclusion:**

This study showed that ventilation during mechanical cardiopulmonary resuscitation using mechanical chest compression devices was efficient with both ventilation strategies. Interestingly, the respiratory minute ventilation volume was even higher with continuous ventilation than with 30:2 due to the higher respiratory rate. In addition, there was no significant difference in ventilation outcome between manual and mechanical chest compressions.

**Supplementary Information:**

The online version contains supplementary material available at 10.1186/s12873-026-01493-z.

## Introduction

Out-of-hospital cardiac arrest affects 380,000 people in the US and 270,000 people in Europe each year. Overall survival rates vary from 8% to 12% [1–3] with favorable neurological outcomes (CPC 1–2) in high-income countries [4]. Cardiopulmonary resuscitation (CPR) focuses on maintaining circulation and oxygenation in patients with cardiac arrest, with the goal of restoring spontaneous circulation (ROSC) [1, 2].

In recent years, numerous investigations have demonstrated that the quality of chest compressions is a decisive factor for patient outcomes during CPR [5–8]. Mechanical chest compression devices (MCDs) provide a constant force and frequency of chest compressions and enable high-quality CPR during transport. These devices increase cardiac output and therefore cerebral and cardiac perfusion [9]. In contrast, manual chest compressions are often limited and of poor quality. This is especially true when rescues are performed in difficult terrain [10, 11]. Two studies by Putzer et al. and Gässler et al. have shown that mechanical CPR (mCPR) is significantly of higher quality than manual chest compressions during helicopter transport and difficult rescue operations [12, 13]. Thus, the use of MCDs increased more than fourfold from 1.9% to 8.0% in out-of-hospital cardiac arrest patients treated by emergency medical services (EMS) across the United States [14]. However, no survival benefit has been shown for the use of MCDs so far [15] and an optimal invasive ventilation strategy for mCPR has not yet been established.

The European Resuscitation Council (ERC) and the American Heart Association (AHA) recommend that effective ventilation should be provided to cardiac arrest patients. Ventilation during CPR is crucial for adequate oxygen administration and carbon dioxide elimination. For manual CPR, international guidelines recommend uninterrupted chest compressions at a ventilation rate of 10/min once intubation has been achieved [16]. Each breath should take approximately 1 s to inhale and should be sufficient to produce a visible chest rise (500–600 ml) [16, 17]. Maintaining continuous chest compressions is important as small interruptions may have a negative impact on survival and neurological outcome [9].

In the past, studies have mainly focused on chest compressions, with sometimes conflicting results. Perhaps for this reason, previous trials of continuous versus interrupted chest compressions have failed to show a survival benefit for either strategy [18–20]. More recently, studies have focused on different ventilation strategies and their impact on survival. Interestingly, a recent study showed that with good adherence, interrupted CPR (30:2) resulted in better survival rates [19]. In addition, a retrospective study showed that asynchronous ventilation may confer a survival benefit [21]. Conversely, a recent meta-analysis indicated that there is no statistically significant difference between the two strategies with regard to ROSC or favorable neurological outcome [22].

In conclusion, the evidence for recommendations regarding ventilation and oxygenation during different methods of CPR is weak and sometimes conflicting [23, 24]. Particularly, there are no clear ventilation recommendations for mCPR. Some experts question the effectiveness of continuous ventilation (CV) due to reduced ventilatory parameters measured by ventilators [25]. We aimed to investigate differences in respiratory minute ventilation volume between manikins resuscitated manually and those treated with a mechanical chest compression device. We hypothesized that the method of chest compressions would influence ventilation parameters, potentially leading to measurable differences in respiratory minute ventilation volume.

## Material und methods

### Design and setting

This prospective experimental study at the University Hospital of Innsbruck (Austria) compared two ventilation strategies (continuous ventilation versus 30:2) during mCPR (with different MCDs) and manual CPR. CV was performed with uninterrupted chest compressions (target range 105/min) and a ventilation rate of 10 ventilation breaths per minute (1 breath every 6 s). In the 30:2 strategy, chest compressions were interrupted by 2 ventilation breaths after every 30 compressions.

### Randomisation

Sample size calculation was performed using G*Power (Version 3.1.9.7) for a Mann-Whitney U test, assuming an alpha error of 0.05, a power of 0.90, and an effect size of 0.75 based on an assumed mean difference of 0.5 l in respiratory minute ventilation volume. Accounting for an anticipated dropout rate of 10%, the calculated sample size was 90 cases, which were required to ensure sufficient statistical power for reliable results. Thirty medical students were randomized to one of two mechanical compression devices (LUCAS^®^ or EASY PULSE^®^; 15 students per group). For the manual CPR trials, students were paired into teams of two. Each student performed one mechanical and one manual (team-based) CPR session under both continuous chest compression and 30:2 compression-to-ventilation conditions, resulting in a total of 90 resuscitations—45 with continuous chest compressions and 45 following a 30:2 compression-to-ventilation ratio. The study design and randomization process are illustrated in the accompanying flowchart (Electronic supplemental material Fig. 1). Every CPR scenario was performed for 3 min. 15 participants used the LUCAS^®^ and 15 participants used the EASY PULSE^®^ as MCD. Manual CPR was performed in teams of two persons and chest compressions were switched after 1.5 min so that each participant had to ventilate equally. In total, 90 tests were performed of which 4 had to be excluded because of technical issues. Every participant had experience in basic and advanced life support and was blinded for the manikin monitors.

### Materials

Following 3 CPR manikins were used: Manikin 1: AmbuMan Advanced, Prod. No. A 284 407 000, Ambu, Ballerup, Dänemark; Manikin 2: AmbuMan Airway Wireless, Prod. No. A 284 427 000, Ambu, Ballerup, Dänemark; Manikin 3: Ambu M MegaCode W, Prod. No. A 284 307 000, Ambu, Ballerup, Dänemark. The resistance for chest compressions was set to “medium” (8.5 Nm). All three manikins used in this study were constructed identically with respect to airway anatomy and thoracic structure. The only differences between the models were peripheral parts such as arms and feet, which do not influence airway or chest mechanics. The maximum ventilation volume for all three manikins was 1.2 l.

Two different MCD systems were used: LUCAS^®^ 2 (Physio-Control, Redmond, USA) and EASY PULSE^®^ (Schiller Medizintechnik GMBH, Feldkirchen, Germany). The collected data was documented in Microsoft Excel (Version 16.70, Microsoft Corporation, Redmond, USA).

All manikins were endotracheally intubated using a Super Safety Clear Flexiset endotracheal tube (inner diameter 8.0 mm, Teleflex Medical, Ireland). Ventilation during cardiopulmonary resuscitation was performed manually using a bag-valve mask (Ambu^®^ SPUR II, Ambu A/S, Ballerup, Denmark). Ventilation parameters, frequency, compression depth and incorrect hand position were recorded by the manikins.

### Data collection and statistical analysis

During CPR the following data was recorded by the manikins:

- Respiratory minute volume ventilation (l/min),
- respiratory rate (f/min),
- tidal volume (ml),
- depth of chest compressions (mm),
- ratio of chest compression and unloading (% [“duty cycle”]),
- wrong position of the hands (total number),
- leaning/chest recoil quantity.

The primary endpoint of the study was the respiratory minute ventilation volume during CPR. Secondary endpoints included the differences in respiratory minute ventilation volume between the (CV) and 30:2 compression-to-ventilation rhythm when using mechanical compression devices and during manual chest compressions, as well as the differences between the two ventilation rhythms for each mechanical compression device separately. In addition, correlations between tidal volume (TV) and compression depth were analyzed.

The data were analyzed using the Shapiro–Wilk test to assess normal distribution. As none of the variables were normally distributed, group comparisons were performed with the Mann–Whitney U test, and correlations were assessed using the Pearson correlation coefficient.

A Pearson correlation analysis between compression depth and TV was conducted for both CPR methods with CV and 30:2 rhythm. A two-sided p-value of less than 0.05 was considered statistically significant.

## Results

This study included 86 CPRs performed by 30 medical students. All students were trained in basic and advanced life support. The median age of the student was 24 (IQR: 23–24) and 60% were female. All participants were able to complete the tests correctly (see Table 1).

**Table 1:** Baseline and ventilation data

**Students**			
Age	24 (23–24)		
Male	18 (37.9%)		
Medical trained	12 (41.4%)		
**Overall**	**Continuous**	**30:2**	**p**
Respiratory minute ventilation volume (l/min)*	4.80 (4.10–5.40)	3.10 (2.80–3.40)	< 0.001
Ventilation frequency (/min)	7.60 (5.90–8.60)	4.20 (3.80–4.90)	< 0.001
Tidal volume (ml)*	521.00 (484.00–561.00)	565.00 (503.00–600.00)	0.013
Compression Frequency (/min)*	100.30 (99.40–102.00)	100.00 (86.00–102.00)	0.019
Compression depth (mm)*	51.00 (34.70–56.30)	48.00 (34.00–57.40)	0.832
Work performed per minute (mm/min)*	5304.00 (3483.88–5849.89)	4568.40 (3200.00–5457.00)	0.103
Wrong handposition (number)*	0 (0–1)	0 (0–2)	0.678
Ratio of chest compression and unloading (%)*	1.00 (0.98–1.00)	0.90 (0.89–0.91)	< 0.001
*Pearson correlation*: Tidal volume – Compression Depth	− 0.210	0.185	
**Manual**	**Continuous**	**30:2**	
Respiratory minute ventilation volume (l/min)*	4.80 (4.30–5.10)	3.05 (2.90–3.30)	< 0.001
Ventilation frequency (/min)	7.80 (6.00–8.00)	4.15 (3.90–4.80)	< 0.001
Tidal volume (ml)*	511.00 (500.00–552.00)	580.00 (550.00–603.00)	0.004
Compression Frequency (/min)*	103.10 (98.00–115.00)	94.85 (90.00–102.00)	0.012
Compression depth (mm)*	54.30 (50.40–61.10)	56.90 (45.90–61.00)	0.946
Work performed per minute (mm/min)*	5891.35 (5141.00–6135.44)	5421.54 (4344.27–5917.00)	0.104
Wrong handposition (number)*	1.00 (0.00–97.00)	2.00 (0.00–52.00)	1
Ratio of chest compression and unloading (%)*	0.97 (0.95–0.98)	0.89 (0.89–0.90)	< 0.001
*Pearson correlation*: Tidal volume – Compression Depth	− 0.119	0.247	
**Mechanical**	**Continuous**	**30:2**	
Respiratory minute ventilation volume (l/min)*	4.60 (4.10–5.50)	3.10 (2.80–3.40)	< 0.001
Ventilation frequency (/min)	7.40 (5.40–9.00)	4.30 (3.80–4.90)	< 0.001
Tidal volume (ml)*	525.00 (458.00–566.00)	541.00 (500.00–593.00)	0.228
Compression Frequency (/min)*	100.30 (99.90–102.00)	100.40 (85.80–101.90)	0.472
Compression depth (mm)*	41.10 (32.10–54.80)	38.20 (30.80–54.00)	0.780
Work performed per minute (mm/min)*	4122.33 (3206.79–5589.60)	3835.28 (2918.90–4962.53)	0.208
Wrong handposition (number)*	0	0	0.666
Ratio of chest compression and unloading (%)*	1.00 (1.00–1.00)	0.90 (0.90–0.91)	< 0.001
*Pearson correlation*: Tidal volume – Compression Depth	− 0.246	0.081	

*: median (IQR)

### Ventilation

**Fig. 1:**
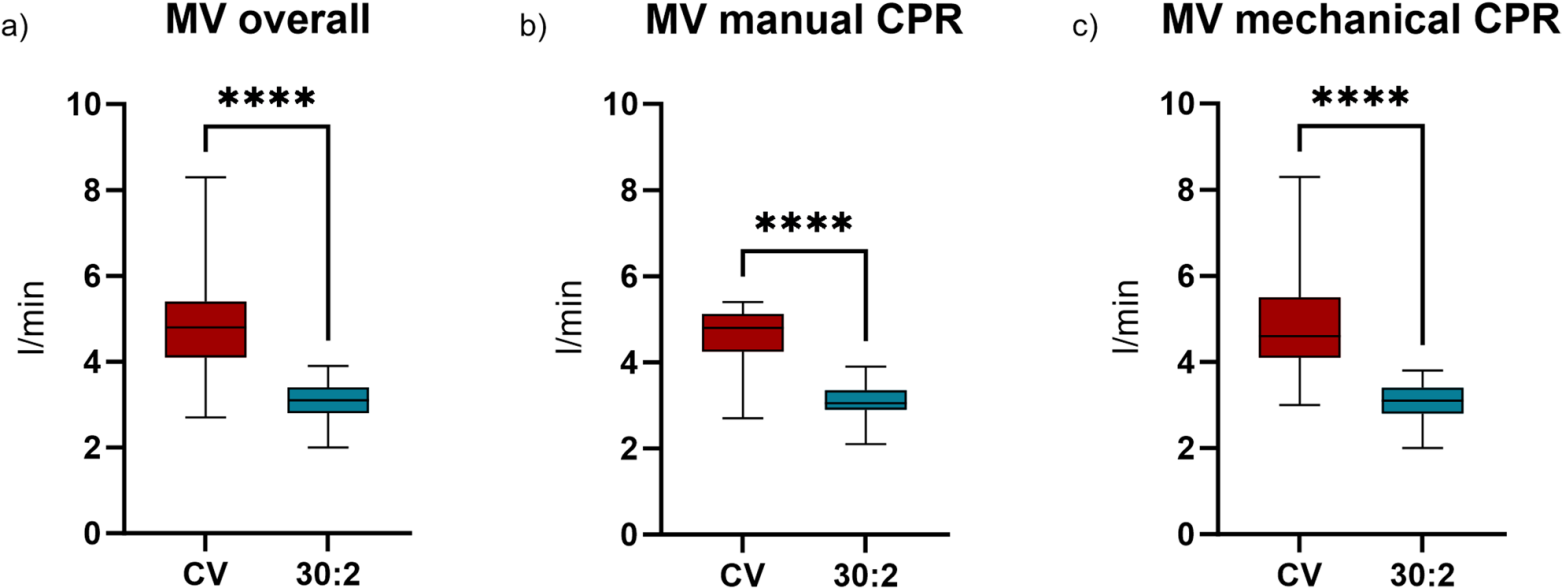
Measured respiratory minute ventilation volume (**a**) overall, (**b**) while performing manual chest compression and (**c**) while using a mechanical chest compression device

**Fig. 2:**
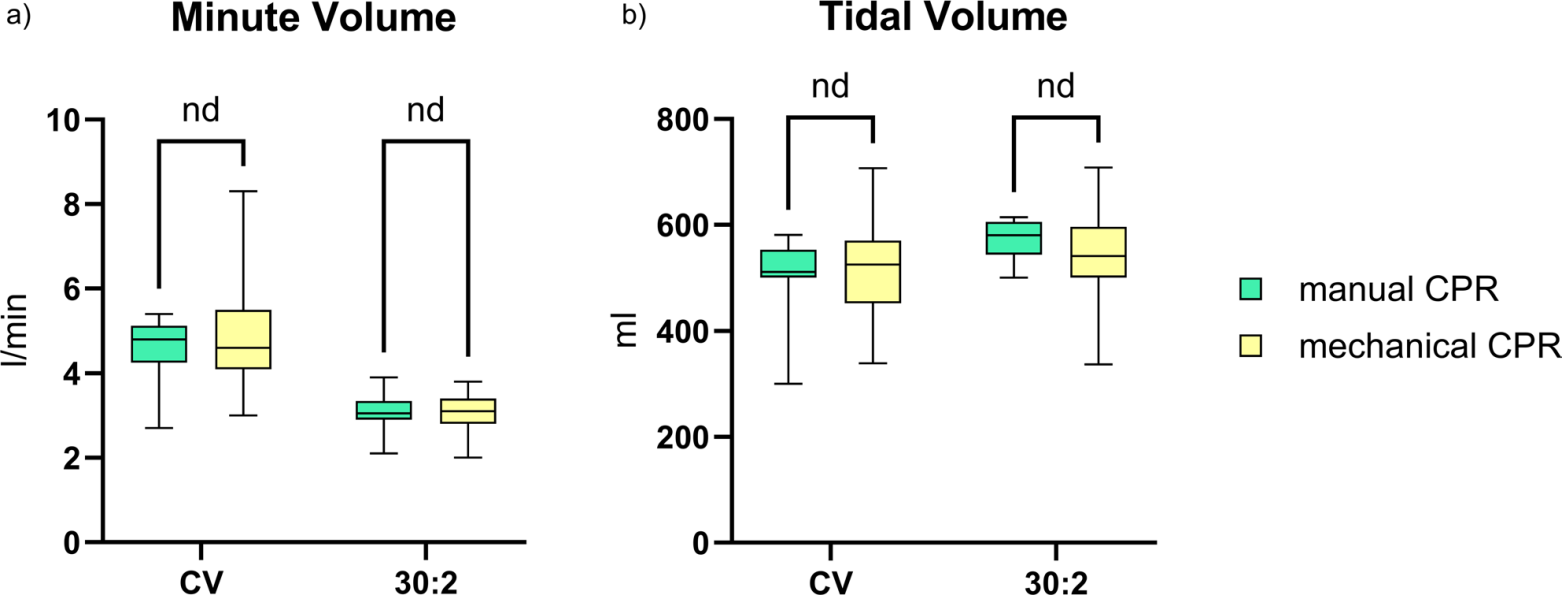
Comparison of (**a**) respiratory minute ventilation volume and (**b**) tidal volume between performing manual chest compressions and using a mechanical chest compression device

Respiratory minute ventilation volume was significantly higher in all trials with CV (4.8 l/min) than with 30:2 CPR (3.1 l/min, *p* < .001, Fig. 1a). In detail, during manual CPR respiratory minute ventilation volume was 4.8 l/min and 3.05 l/min and during mCPR MV was 4.6 l and 3.1 l/min with CV and 30:2 respectively (Fig. 1b-c). Interestingly, there was no significant difference between mechanical and manual CPR with both ventilation rhythms (Fig. 2a).

**Fig. 3:**
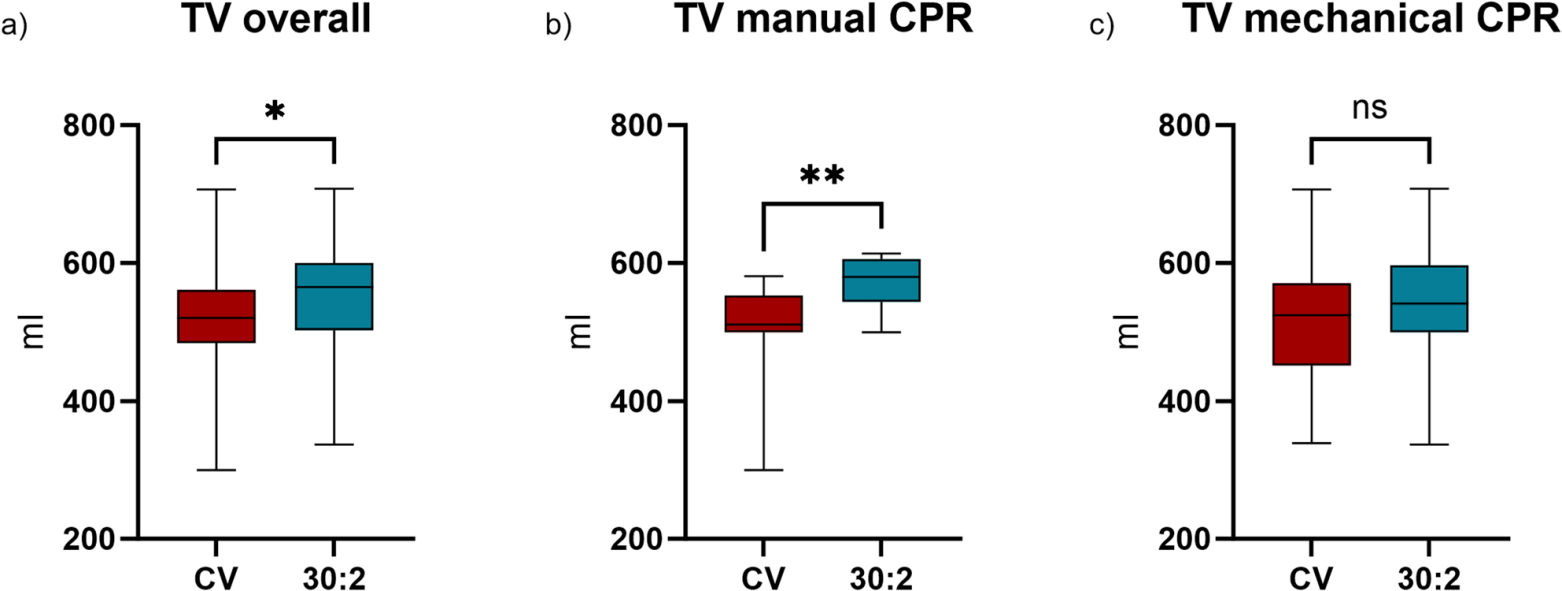
Measured tidal volume (**a**) overall, (**b**) while performing manual chest compressions and (**c**) while using a mechanical chest compression device

TV was lower with CV (521 ml [IQR: 484.0–561.0]) than with 30:2 (565 ml [IQR: 503.0–600.0]; *p*=.013, Fig. 3a). In detail, during manual CPR TV was 511 ml [IQR: 500.0–552.0] and 580 ml [IQR: 550.0–603.0] (*p*=.004) and during mCPR TV was 525 ml [IQR: 458.0–566.0] and 541 ml [IQR: 500.0–593.0) (*p*=.228) with CV and 30:2 respectively (Fig. 3b-c). Despite the lower TV with CV, the respiratory minute ventilation volume was ultimately higher due to the higher ventilation frequency (Table 1). There was no significant difference between mechanical and manual CPR with both ventilation rhythms (Fig. 2b).

A significant difference was also observed in ventilation frequency between the two resuscitation rhythms. The ventilation frequency was 7.6/min [IQR: 5.9–8.6] with CV versus 4.2/min [IQR: 3.8–4.9] with 30:2 rhythm (*p*<.001, Table 1).

### Chest compression

The compression frequency was overall significantly higher with CV (100.3/min [IQR: 99.4–102.0]) than with 30:2 ventilation (100/min [IQR: 86.0–102.0]; *p*=.019, Table 1). The difference was particularly clear in manual CPR (103.1/min [IQR: 98.0–115.0] vs. 94.9/min [IQR: 90.0–102.0], *p*=.012).

The compression depth was median 32.1 mm [IQR: 29.3–35.6] and 30.8 mm [IQR: 29.3–34.5] when using EASY PULSE^®^, 54.8 mm [IQR: 52.3–57.9] and 54.2 mm [IQR: 52.8–57.4] with LUCAS^®^, and 54.3 mm [IQR: 50.4–61.1] and 56.8 mm [IQR: 45.9–61.0] (CV and 30:2, respectively) with manual chest compressions (Tables 1 and 2). This represents a significant difference between EASY PULSE^®^ and LUCAS^®^ (*p*<.001), as well as EASY PULSE^®^ and manual CPR (< 0.001). Notably, there was no significant difference between LUCAS^®^ and manual chest compressions (*p*=.965).

**Table 2:** Ventilation data of mechanical chest compression devices

**Lucas**	**Continuous**	**30:2**	
Respiratory minute ventilation volume (l/min)*	4.70 (4.10–5.50)	3.10 (2.80–3.50)	< 0.001
Ventilation frequency (/min)	7.30 (5.30–8.40)	4.10 (3.80–4.40)	< 0.001
Tidal volume (ml)*	529.00 (435.00–561.00)	552.00 (501.00–600.00)	0.178
Compression Frequency (/min)*	102.00 (99.30–102.00)	101.95 (85.80–102.20)	0.804
Compression depth (mm)*	54.90 (52.30–57.90)	54.20 (52.80–57.40)	0.603
Work performed per minute (mm/min)*	5594.70 (5334.60–5749.47)	5099.97 (4920.00–5508.00)	0.019
Wrong handposition (number)*	0	0	0.804
Ratio of chest compression and unloading (%)*	1.00 (1.00–1.00)	0.91 (0.90–0.92)	< 0.001
*Pearson correlation*: Tidal volume – Compression Depth	− 0.328	− 0.007	
**EasyPulse**	**Continuous**	**30:2**	
Respiratory minute ventilation volume (l/min)*	4.60 (4.10–5.50)	3.10 (2.70–3.40)	< 0.001
Ventilation frequency (/min)	7.60 (5.40–9.00)	4.50 (3.10–5.00)	< 0.001
Tidal volume (ml)*	521.00 (500.00–583.00)	541.00 (500.00–593.00)	0.744
Compression Frequency (/min)*	100.10 (99.90–100.30)	100.00 (85.40–100.40)	0.838
Compression depth (mm)*	32.10 (29.30–35.60)	30.80 (29.30–34.50)	0.806
Work performed per minute (mm/min)*	3206.79 (2932.93–3574.24)	2918.70 (2750.96–3413.60)	0.217
Wrong handposition (number)*	0	0	0.967
Ratio of chest compression and unloading (%)*	1.00 (1.00–1.00)	0.90 (0.90–0.90)	< 0.001
*Pearson correlation*: Tidal volume – Compression Depth	− 0.085	0.108	

*: median (IQR)

The main difference between EASY PULSE^®^ and the other chest compression methods was the depth of compression. Therefore, a correlation analysis was performed between TV and compression depth. We found no correlation with 30:2 rhythm, but there was a tendency to a negative correlation with CV, especially during LUCAS^®^ CPR (Tables 1 and 2).

### Ventilation with mechanical chest compression devices

In order to examine respiratory minute ventilation volume and TV in more detail, the results for mCPR with LUCAS^®^ and EASY PULSE^®^ were analyzed separately.

**Fig. 4:**
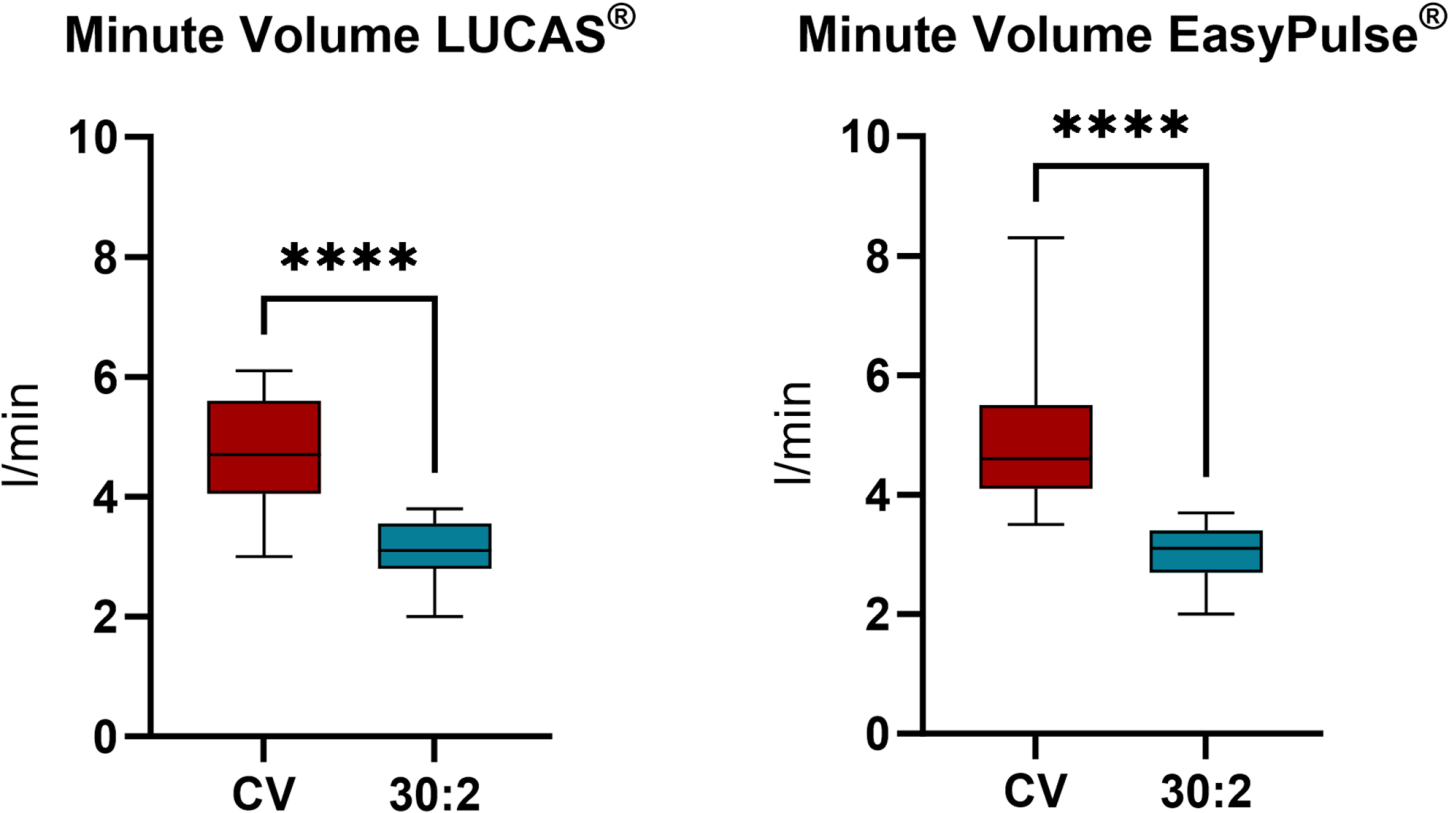
Comparison of respiratory minute ventilation volume between continuous ventilation and 30:2 rhythm with (**a**) LUCAS^®^ and (**b**) EasyPulse^®^

MV was 4.8 l/min [IQR: 4.3–5.1] and 3.1 l/min [IQR: 2.9–3.3] for CV and 30:2, respectively, using LUCAS^®^ and 4.6 l/min [IQR: 4.1–5.5] and 3.1 l/min [IQR: 2.8–3.4], respectively, with EASY PULSE^®^ (Fig. 4, *p*<.001).

**Fig. 5:**
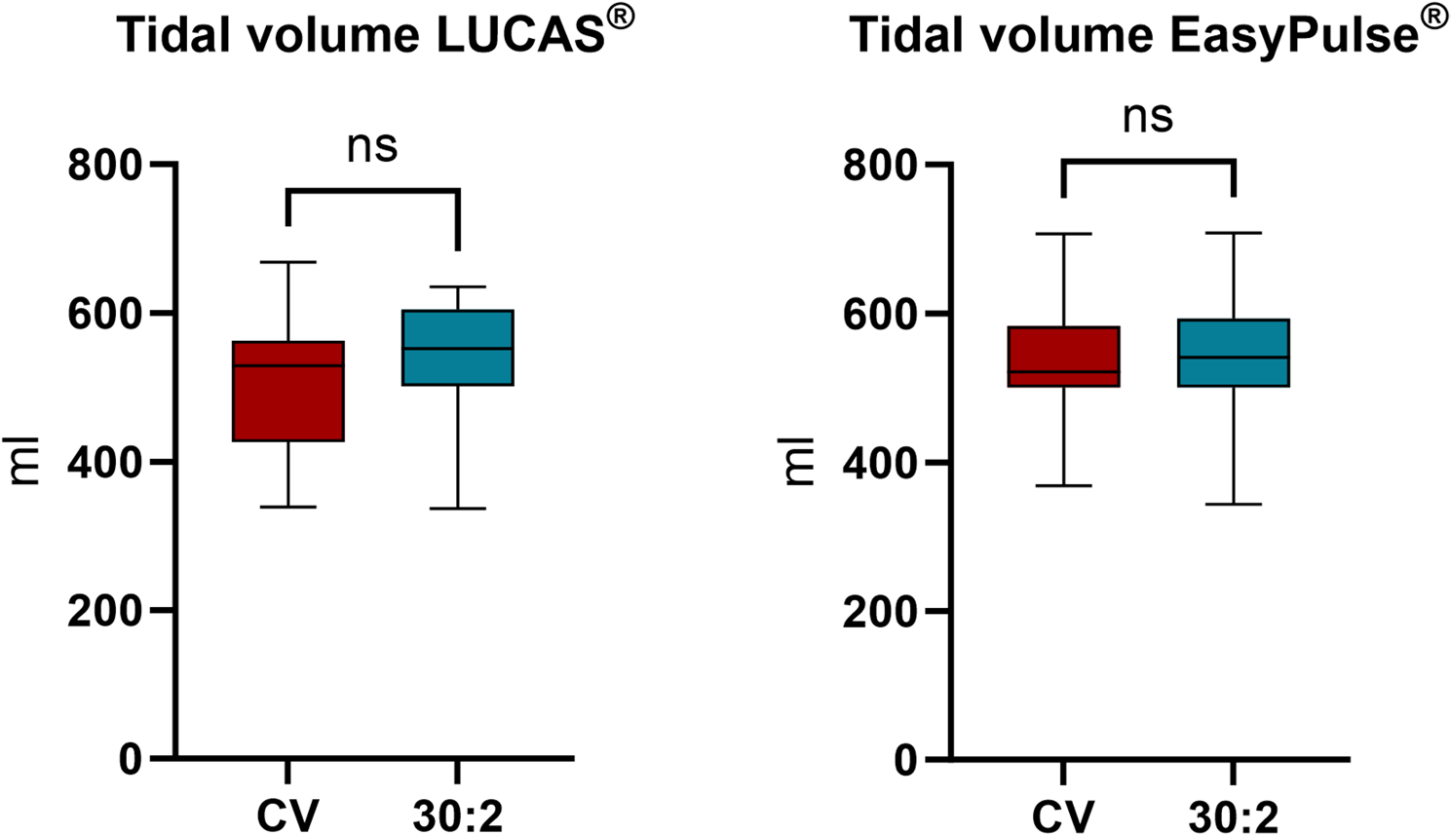
Comparison of tidal volume between continuous ventilation and 30:2 rhythm with (**a**) LUCAS^®^ and (**b**) EasyPulse^®^

TVs were 529 ml [IQR: 435.0–561.0] and 552 ml [IQR: 501.0–600.0] with LUCAS^®^ and 521 ml [IQR: 500.0–583.0] and 541 ml [IQR: 500.0–593.0] with EASY PULSE^®^ (CV and 30:2 respectively, Fig. 5; Table 2). Ventilation frequency, compression frequency, compression depth and work performed per minute were higher in both MCDs with CV than with 30:2 (Table 2).

## Discussion

This study compared the two common ventilation strategies (continuous vs. 30:2) during manual and mCPR, and their impact on ventilation. The existing hypothesis suggests that mechanical ventilation with CV should be lower during mCPR with MCDs [25]. Due to the constant high pressure during mechanical chest compressions, ventilation is assumed to remain less effective, partly due to the reversed airflow [26]. However, our study shows that both ventilation strategies provide sufficient ventilation during manual as well as mCPR. An explanation for the discrepancy between the previous publications and our findings could be a fluctuating display of the TV on some ventilators, which may lead to the misconception of ineffective ventilation.

In our study the average ventilation frequency was significantly higher with CV than with 30:2. Therefore, it is likely that the higher MV is attributable to the higher ventilation frequency in this group. These values fall below the recommended ventilation frequency of 10/min, which was achieved in only 7% of the attempts. This recommendation, however, is based on very limited evidence [27], drawn from animal studies, where hyperventilation has been linked to increased intrathoracic pressure, decreased coronary and cerebral perfusion, and decreased ROSC [28, 29]. A systematic review by Vissers et al. revealed no significant differences in survival and favourable neurological outcomes between ventilation rates > 10/min and < 10/min [27].

When analysing TV, a significantly larger (albeit clinically not significant) TV was observed with the 30:2 rhythm compared to CV. In order to investigate this question in more detail, a correlation analysis was performed between compression depth and TV. The negative relationship between compression depth and TV can be explained by several considerations. First, continuous chest compressions reduce the volume of the thorax. When the thoracic volume is restricted, the lungs are unable to expand effectively through external ventilation. Second, continuous chest compressions create a positive pressure in the alveolar and bronchial spaces, leading to a negative airflow and air trapping [26, 30, 31]. Third, greater compression depth increases pressure on the thorax and airways, requiring a higher ventilation pressure. In summary, it can be postulated that a greater compression depth during CPR increases the pressure on the thorax, resulting in a lower ventilation efficiency.

Notably, during the use of the MCD EASY PULSE^®^, no significant difference in TV was found between the two different resuscitation strategies. Further investigations revealed that the average compression depth was only 32 mm with EASY PULSE^®^, which is below the compression depth recommended by the guidelines [16, 17]. One reason for this difference in compression depth, and therefore MCD efficiency, could be the use of different compression technologies. LUCAS^®^, similar to hands-on chest compressions, relies on a piston technique, while EASY PULSE^®^ uses a combination of belt and piston techniques.

There is a strong association between compression depth and the likelihood of ROSC, 1-day survival, and survival until hospital discharge reported in previous studies [32]. A study by Stiell et al. also demonstrated, that a greater compression depth is associated with a higher probability of survival. The highest survival rate was achieved with a compression depth in the range of 40.3 to 55.3 mm, with a peak at 45.6 mm. Notably, survival probability decreased sharply when the compression depth was less than 40 mm [33]. Egger et al. obtained similar results in their study. They examined the effectiveness of various MCDs in alpine environments and found that the compression depth was 65.9 mm with LUCAS^®^ and only 40.2 mm with EASY PULSE^®^ [10].

In another study Ryu et al. compared the compression depth and the resulting blood flow in pigs between LUCAS^®^ 2 and EASY PULSE^®^. Although in Ryu et al.’s study the compression depth with EASY PULSE^®^ was also quite low with 32.8 mm, the systolic diameter of the femoral artery, blood flow, and EtCO2 were higher than with LUCAS^®^ 2. The authors attributed these results to the combination of belt and piston techniques used by EASY PULSE^®^, which could lead to higher intrathoracic pressure. However, there are some limitations to this study that restrict the significance of these results, since the study was conducted in a swine model using a small sample size [34].

To date, there have been no clinical studies on human subjects that have successfully demonstrated the efficacy of mechanical chest compression with the EASY PULSE^®^ device. In addition, EASY PULSE^®^ could not meet the AHA recommendations regarding compression depth. Therefore, there is a compelling rationale for the conduct of clinical studies on human subjects to evaluate the effectiveness of EASY PULSE^®^ CPR.

### Limitations

This experimental study investigated the effects of different CPR rhythms on manikins. The manikin’s sensors are capable of precisely recording physical measurements such as compression depth, frequency and volume parameters of ventilation. On the other hand our study designs lacks he capability to record physiological metrics as blood flow, etCO2, or oxygenation and decarboxylation, as well as biologic variations of patients requiring CPR cannot be recorded in this study design. However, by using manikins, we were able to establish reproducible conditions, allowing for an objective comparison between the different strategies. It is unknown to what extent the results can be applied to human bodies and thus should be investigated in a follow-up study.

## Conclusion

Ventilation during mCPR is considered efficient with both ventilation strategies (continuous and 30:2) according to ERC/AHA guidelines. Due to the higher frequency of ventilations, the respiratory minute ventilation volume is even higher with CV than with 30:2. However, the TV presents an inverse picture. TV is slightly but significantly smaller in CV compared to 30:2, presumably due to the consistently high pressure on the thorax. Nevertheless, an average TV of over 500 ml could be achieved in both rhythms, which suggests that the minor discrepancy may not be clinically significant. mCPR with LUCAS^®^ and manual chest compressions achieved high-quality results and adhered to AHA guidelines. However, according to our data, EASY PULSE^®^ could not meet the AHA recommendations regarding compression depth and should be re-evaluated in further studies.

## Supplementary Information

Below is the link to the electronic supplementary material.

Supplementary Material 1

## Data Availability

The datasets used and/or analysed during the current study are available from the corresponding author on reasonable request.
