# Supplementary material for: Continuous ventilation versus 30:2 strategy in mechanical cardiopulmonary resuscitation – a manikin-based simulation study
Source: BMC Emerg Med. 2026 Feb 11;26:74. doi: 10.1186/s12873-026-01493-z (PMC12998201; doi:10.1186/s12873-026-01493-z)
Supplement: Supplementary file 1 — Supplementary Material 1 [file 12873_2026_1493_MOESM1_ESM.docx]

Electronic supplemental material

**Continuous ventilation versus 30:2 strategy in mechanical cardiopulmonary resuscitation – an in vitro study**

Bernhard Benda^1^, Oliver Fuchs^2^, Magdalena Benda^3^, Fabian Perschinka^1^, Nicolas Prokes^1^, Thomas Ploner^1^, Andrea Köhler^1^, Michael Joannidis^1*^ and Frank Hartig^1*^

^1^ Core facility Internal Emergency and Intensive Care Medicine, University Clinic Innsbruck, Innsbruck, Austria

^2^ Department of Internal Medicine, Kufstein Hospital, Kufstein, Austria

³ Department of Internal Medicine II, Academic Teaching Hospital, Feldkirch, Austria

*Corresponding Authors: Frank Hartig, Michael Joannidis

Bernhard.Benda@tirol-kliniken.at

Fuchs.oe@icloud.com

Magdalena.Benda@lkhf.at

Fabian.perschinka@i-med.ac.at

Nicolas.Prokes@tirol-kliniken.at

Thomas.Ploner@tirol-kliniken.at

Andrea.Koehler@i-med.ac.at

Michael.Joannidis@i-med.ac.at*, Tel.: 0043 512 504 24181, Fax: 0043 512 504 24196

Frank.Hartig@tirol-kliniken.at*, Tel.: 0043 512 504 27057


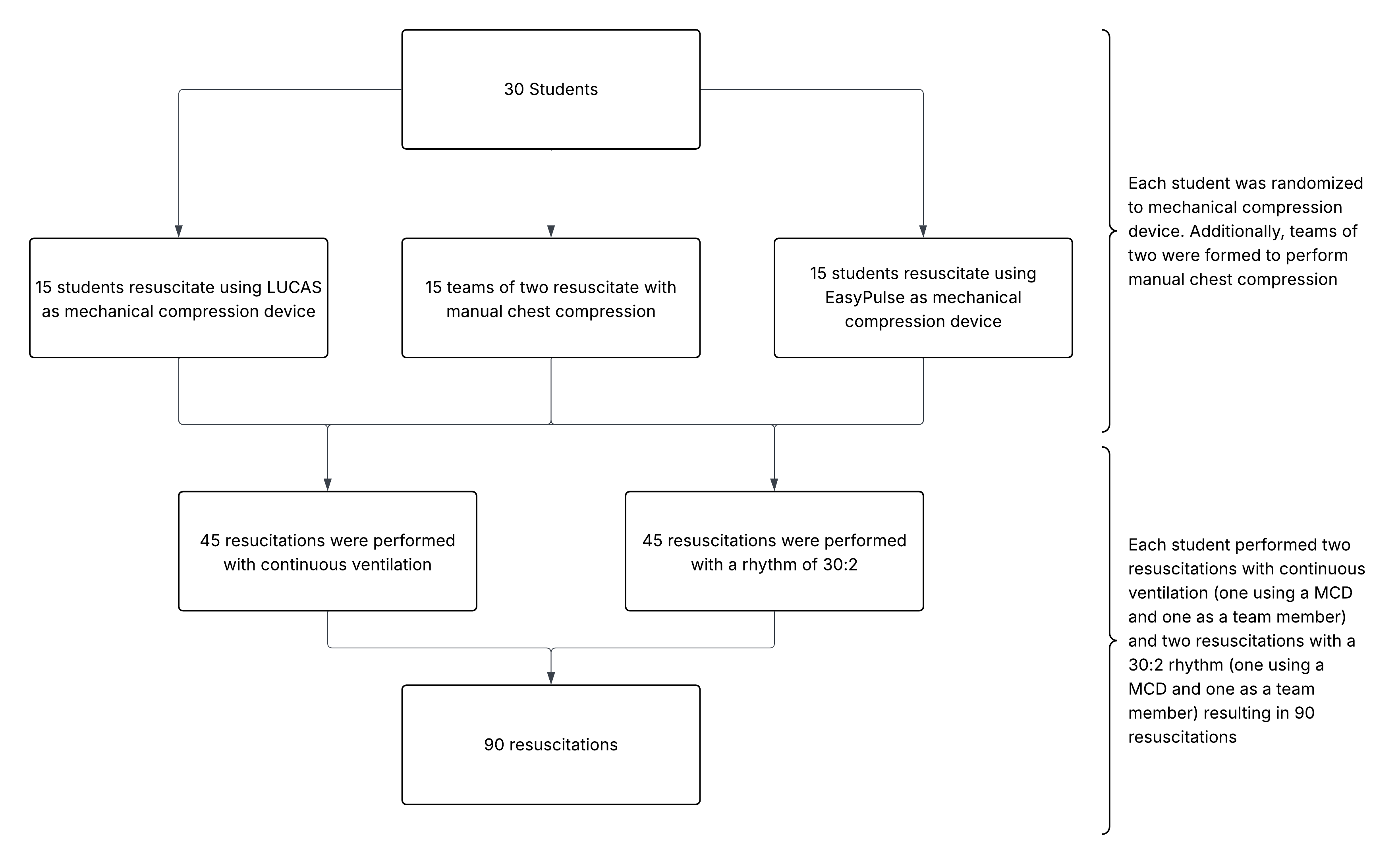


ESM Figure 1: Randomisation flowchart
